# Comparing the Impact of Heart Rate-Based In-Game Adaptations in an Exergame-Based Functional High-Intensity Interval Training on Training Intensity and Experience in Healthy Young Adults

**DOI:** 10.3389/fpsyg.2021.572877

**Published:** 2021-06-21

**Authors:** Anna Lisa Martin-Niedecken, Tiziana Schwarz, Alexandra Schättin

**Affiliations:** ^1^Subject Area in Game Design, Department of Design, Zurich University of the Arts, Zurich, Switzerland; ^2^Motor Control and Learning, Institute of Human Movement Sciences and Sport, Department of Health Sciences and Technology, ETH Zürich, Zurich, Switzerland

**Keywords:** exergame, game balancing, heart rate, effectiveness, attractiveness, assessement, ExerCube, in-game adaptation

## Abstract

Physical inactivity remains one of the biggest societal challenges of the 21st century. The gaming industry and the fitness sector have responded to this alarming fact with game-based or gamified training scenarios and thus established the promising trend of exergaming. Exergames—games played with the (whole) body as physical input—have been extolled as potential attractive and effective training tools. Simultaneously, researchers and designers are still exploring new approaches to exploit the full potential of this innovative and enjoyable training method. One way to boost the attractiveness and effectiveness of an exergame is to individualize it with game adaptations. A physiological parameter that is often used to balance the physical challenge and intensity of exergames to the player’s fitness skills is the heart rate (HR). Therefore, researchers and designers often rely on age-based, maximum HR (HR_max_) formulas originating from performance diagnostics. In combination with the player’s assessed real-time HR during an exergame session, the pre-determined HR_max_ is used to adapt the game’s challenge to reach a pre-defined HR and physical intensity level (in-exergame adaptations), respectively. Although the validity and reliability of these age-based HR_max_ formulas were proven in heterogeneous target populations, their use is still often criticized as HR is an individual parameter that is affected by various internal and external factors. So far, no study has investigated whether the formula-based pre-calculated HR_max_ compared to a standardized individually pre-assessed HR_max_ elicits different training intensities, training experiences, and flow feelings in an exergame. Therefore, we compared both variants for in-exergame adaptation with the ExerCube – a functional high-intensity interval training exergame – in healthy young adults. Comparing the results of the two conditions, no significant differences were found for HR parameters and perceived physical and cognitive exertion, nor for overall flow feelings and physical activity enjoyment. Thus, the formula-based in-exergame adaptation approach was suitable in the presented study population, and the ExerCube provided an equally reliable in-exergame adaptation and comparable exergame play experiences. We discuss our findings in the context of related work on exergame adaptation approaches and draw out some implications for future adaptive exergame design and research topics.

## Introduction

Numerous guidelines preach the urgent necessity of regular physical activity to maintain a physically and mentally healthy lifestyle at all ages. According to the American College of Sports Medicine, a program of regular exercise, including cardiorespiratory, resistance, flexibility, and neuromotor training in addition to the activities of daily living, is essential for most adults to improve and maintain their physical fitness and health (Garber et al., 2011). However, surveys by the World Health Organization continuously reveal that physical inactivity remains the greatest public health problem of the 21st century (Trost et al., 2014). In addition to a lack of motivation and time, changing behavioral and environmental factors as well as a number of common exercise barriers are the main reasons for this persisting problem (Trost et al., 2002). Therefore, stakeholders from various fields have called for new concepts for attractive and effective training alternatives to reduce entry barriers and help to maintain training adherence for a wide range of people over a period of several years (Marshall and Linehan, 2020).

Exergames, which require physical effort and are controlled by (whole) body movements (Oh and Yang, 2010), have been promoted as suitable tools for providing attractive and effective training alternatives or supplements by the interdisciplinary research and development (R&D) community. In more than 10 years, R&D work has proved that exergames have the potential to be training tools that are both effective (i.e., increasing physical-cognitive fitness, endurance, strength, and coordination) (Staiano and Calvert, 2011a,b; Sween et al., 2014; Best, 2015; Benzing et al., 2016; Kari, 2017; Mura et al., 2018; Stojan and Voelcker-Rehage, 2019; Xiong et al., 2019) and attractive (e.g., increasing training adherence, motivation, flow, and engagement) (McRae et al., 2012; Valenzuela et al., 2018; Martin-Niedecken et al., 2019b; Tondello et al., 2019). Furthremore, exergamging promotes physical activity and training in different target populations (Lu et al., 2013; Kappen et al., 2019; Martin-Niedecken and Schättin, 2020). However, the majority of the evaluated exergames, which were not necessarily specifically designed for the purpose of obtaining certain training results or to be used as a motivating alternative to traditional training methods, did not meet the required intensity or effectiveness, nor did they induce the intended training adherence or long-term motivation (e.g., Marshall and Linehan, 2020).

A promising approach towards boosting the effectiveness (e.g., training intensity and outcomes) and attractiveness (e.g., flow, immersion, enjoyment, and motivation) of exergames is the personalization through system adaptations, also known as dynamic game balancing (Mueller et al., 2012; Altimira et al., 2016), game difficulty adjustment (Adams, 2010), dynamic difficulty adjustments (Hunicke, 2005), or multiplayer game balancing (Gerling et al., 2014). Besides various pre-exergame and real-time in-exergame adaptation parameters (e.g., game speed, frequency of in-game tasks, and increasing input-movement intensity and range), in-exergame adaptations based on the player’s heart rate (HR) have been proposed and explored by various researchers and designers. These HR-based concepts were proven to be feasible and beneficial approaches to balance the player’s abilities and the challenges of the exergame (Sinclair et al., 2009; Stach et al., 2009; Mueller et al., 2012; Hoffmann et al., 2015; Ketcheson et al., 2015; Martin-Niedecken and Götz, 2016, 2017; Martin-Niedecken, 2018; Martin-Niedecken and Mekler, 2018; Muñoz et al., 2018; Martin-Niedecken et al., 2019a,b). At the same time, these formulas are often criticized because HR is an individual parameter that is influenced by various internal and external factors (Stach et al., 2009; Mueller et al., 2012; Hoffmann et al., 2015; Ketcheson et al., 2015). However, no study has yet investigated whether the formula-based pre-calculated maximum HR (HR_max_) compared to a standardized individually pre-assessed HRmax elicits different training intensities, training experiences, and flow feelings in an exergame. Furthermore, exergames and their underlying technologies have the potential to serve as assessment tools for in-exergame adaptation parameters, and could thus replace strenuous and unmotivating traditional test procedures with playful assessments (e.g., Konstantinidis et al., 2015). Nonetheless, this has not yet been explored in much detail. To sum up, further interdisciplinary R&D work is needed to fill these gaps and to further explore the full potential of (adaptive) exergames as an innovative training and assessment tool.

Our work explores these R&D gaps, with the goal of better understanding the design requirements and potential of attractive and effective exergames. Based on an overview of different pre- and in-exergame adaptation approaches, we present a study that compares two different play conditions of a newly developed, adaptive functional high-intensity interval training (fHIIT) exergame, the so-called ExerCube, using objective and subjective measures for training intensity (effectiveness) and experience (attractiveness) in healthy young adults. The ExerCube automatically adapted to the targeted HR range (defined as percentage of HR_max_) by either (i) the individually pre-assessed HR_max_ or (ii) the formula-based pre-calculated HR_max_. Furthermore, we explored the potential utility of the ExerCube as a HR_max_ assessment tool by comparing the ExerCube procedure to a standard ergometer protocol. Based on our results, we discuss how individual in-exergame adaptation can influence the effectiveness and attractiveness of exergames.

## Related Work

During the last few years, interdisciplinary researchers and designers have started exploring different exergame adaptation approaches in various target populations. These approaches were based on theories and models from disciplines such as human movement science, human–computer interaction (HCI), game research, and psychology.

### Multi-Level Pre-exergame Adaptations

Pre-exergame adaptations based on various design levels of an exergame have so far been considered only by a limited number of R&D studies. Following, we present selected studies that used pre-exergame adaptations in different application areas.

Hardy et al. (2014) tested the dependence of motivation, perceived difficulty, and performance on specific level features as well as goal-setting in a balance exergame to find out whether the effectiveness of the training can be increased intentionally by changing level features or setting personal goals. They found a significant influence of single features on psychological (motivation and diffculty) and physiological constructs (performance and play time).

Altimira et al. (2013, 2014) investigated how two traditional balancing approaches (i.e., playing with the non-dominant hand and using a pre-determined head-start) affect players’ experiences in traditional table tennis and in game-based table tennis with the Nintendo Wii, respectively. Their studies showed that playing with the non-dominant hand discouraged players in traditional table tennis and that having a score disadvantage discouraged them in the digital version. Building on this, Altimira et al. (2017) studied how digital technology (i.e., altering the sports equipment: playing with a smaller bat-head or a smaller table) can be used as a resource for game balancing in an augmented table tennis exergame. They showed that dynamic adjustments enhanced engagement more than static adjustments.

Jensen and Grønbæk (2016) developed and evaluated the effects of three balancing schemes based on a physical, an implicit-digital, and an explicit-digital approach, implemented into a ball-controlled exergame. They demonstrated that all three game balancing approaches were feasible and gave equal chances to win while all players enjoyed the balanced gameplay.

Gerling et al. (2014) examined different game adjustments such as score multipliers, the precision of the input movements, and the number of movements implemented in a dancing game. They found that obvious game balancing can reduce players’ self-esteem in comparison to hidden game balancing. Score balancing reduced the appearance of extreme performance gaps between players. The adjustment of input movement precision reduced small differences in players’ performances and in asymmetric physical input, e.g., a player in a wheelchair.

Siegel and Smeddinck (2012) examined an approach to dynamic difficulty adjustments in an exergame for Parkinson’s disease using patients’ range of motion as well as movement speed and accuracy as adaptation parameters. They found that this approach was viable and appreciated by therapists. However, the system might benefit from increased flexibility.

### HR-Based In-Exergame Adaptations

Considerably more widespread is R&D work in the area of in-exergame adaptations, especially focusing on the player’s HR.

Mueller et al. (2012) presented an urban jogging system that used HR data and spatialized sound to create an equitable, balanced experience between joggers of different fitness levels and geographical locations. They demonstrated that real-time HR-based balancing positively affected players’ experiences in a remote jogging application because they performed in their own training zone, while still engaging with another person.

Stach et al. (2009) used HR scaling in a multiplayer cycling exergame. Players’ in-exergame performances were based on their effort relative to their fitness level. They demonstrated that HR scaling reduced the performance gap of different fitness levels. Moreover, engagement was not significantly affected during gameplay.

Ketcheson et al. (2015) evaluated HR power-ups to encourage vigorous training intensities in a cycling exergame intervention. This real-time game mechanism provided in-exergame rewards when players reached the targeted HR level (e.g., the avatar may be more powerful). The pedaling was used to control the avatar’s movement while a standard controller was used to navigate the avatar in different directions and to release in-game actions. Researchers concluded that HR power-ups enhanced exertion levels while also increasing players’ enjoyment levels.

Hoffmann et al. (2015) implemented and tested an algorithm that controlled the physical load of an endurance cycling exergame to approach and maintain a pre-defined HR. They used the pedaling frequency (cadence) as a game-controlling parameter and the resistance (Watt) as an adaptive control mechanism. The evaluation indicated that the developed algorithm was a feasible approach for controlling an individual adaptive training load in the cycling exergame.

Muñoz et al. (2018) investigated the effectiveness and attractiveness of a Pong-like floor-projected exergame in older adults. They showed that real-time HR-based in-exergame adaptations (e.g., speed and training zone) increased the time the older adults spent in the recommended exertion level by approximately 40% compared to conventional functional training. Furthermore, this exergame training provided a controlled, safe, joyful, and effective cardiovascular training in older adults.

### Physical–Cognitive In-Exergame Adaptations

There are two exergame approaches that extend the HR-based approach by exploring HR-based (physical) and performance-related (cognitive) in-exergame adaptations. Sinclair et al.’s (2007, 2009) dual flow model proposes certain design strategies to balance players’ gaming (cognitive) and fitness (physical) abilities with the actual required skills to successfully play and enjoy an exergame in real-time. Based on this model, Martin-Niedecken and Götz (2016, 2017), Martin-Niedecken (2018) designed and evaluated the adaptive fitness game environments Plunder Planet for children and ExerCube for adults (Martin-Niedecken and Mekler, 2018; Martin-Niedecken et al., 2019b). Among other things, they experimented with performance-related and physiologically-based real-time in-exergame adaptation mechanics and implemented them as algorithms.

Plunder Planet (Martin-Niedecken and Götz, 2016, 2017; Martin-Niedecken, 2018) is a single- and two-player exergame that can be played in two versions. One variation includes a full-body motion controller providing physical guidance via six big buttons distributed on two racks (height and weight adjustable) on the player’s left- and right-hand side, requiring cognitive and coordinative skills as well as haptic interactions. The other variation includes the gesture-based Kinect sensor, which allows more natural and intuitive input movements and more freedom of movement. In both variations, the player navigates a flying pirate ship along a deserted racing track and has to overcome virtual obstacles and avoid collisions with sandworms. The player’s HR is tracked via a chest strap. The exergame can be manually or automatically adapted in real-time to the player’s physical and cognitive performance. Thus, the speed of the ship and the frequency of virtual obstacles (physical challenge) are increased or decreased based on the player’s HR. The track characteristics (e.g., flatter or curved) and the number of options for overcoming an obstacle (one to three option(s), i.e., hard to easy, respectively) are adapted to the player’s performance (cognitive challenge). A study in children proved the fundamental functionality and usability (attractiveness and effectiveness) of Plunder Planet (Martin-Niedecken and Götz, 2016). Martin-Niedecken and Götz (2017) found that the implementation of adaptive game mechanics provided players with an enjoyable and effective (moderate intensity) exergame experience, and that playing the game with the different controllers resulted in different spatial presence and gameplay experiences depending on the player’s preferences as well as play and sports skills. An advanced study (Martin-Niedecken and Götz, 2017) demonstrated that the adaptive Plunder Planet version was significantly better than the non-adaptive one in relation to game flow, dual flow, motivation, enjoyment, and spatial presence, as well as the children’s physiological responses.

**FIGURE 1:**
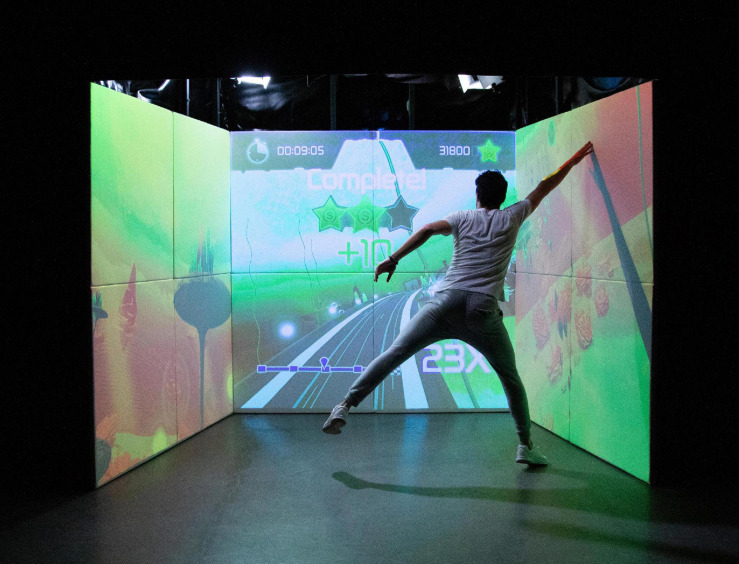
Functional high-intensity interval training in the ExerCube.

The ExerCube (Figure 1; Martin-Niedecken and Mekler, 2018; Martin-Niedecken et al., 2019a,b) is an immersive mixed-reality fitness game for single or multiple players. The player is surrounded by three walls that serve as projection screens and haptic interfaces for energetic bodily interactions. A customized motion-tracking system tracks players’ movements via HTC Vive trackers (attached to their wrists and ankles). In the functional fitness game scenario Sphery Racer, which is projected onto the walls of the ExerCube, the player races along a fast-paced sci-fi underwater race track via an avatar on a hoverboard. The motion-tracking system transfers the executed movements (based on a functional workout) onto the avatar and thus onto the virtual racing track. Along the race, players are challenged by functional whole body exercises (e.g., squats, lunges, and burpees) and by an additional cognitive challenge as players have to quickly process track information and react accordingly (i.e., reaction, planning, and coordination challenges). To ensure an attractive and effective workout experience for a wide spectrum of players with different skill sets, this fitness game continuously adapts game difficulty to players’ individual fitness and cognitive skills. Training intensity is measured via continuous HR tracking (i.e., players wear a HR-sensor chest strap). Depending on the targeted training intensity (e.g., moderate or high), tracking is set to an individually pre-defined HR range, defined as a percentage of HR_max_, where HR_max_ is manually inserted or automatically calculated based on the following formula (Nes et al., 2013):

(1) $$H\,R_{\max}=211-\text{age}\times 0.64$$

Based on this training range and the player’s HR, the exercise difficulty, gaming speed, frequency of obstacles, and track characteristics are increased or decreased within the training session. Cognitive skills are measured by in-exergame performance (reacting to visual stimuli at the right time) and are balanced by the display timing of the next movement direction. In a previous empirical study, the first early stage prototype was found to be on par with personal training in terms of immersion, motivation, and flow (Martin-Niedecken et al., 2019b). A second study with the redesigned prototype aimed to compare the objective and subjective physiological training intensity in the ExerCube to that induced by conventional fHIIT (Martin-Niedecken et al., 2020). This study demonstrated that the ExerCube is a feasible tool for inducing fHIIT intensity. While the average HR was significantly lower than in the conventional functional HIIT condition, maximal HR was at the same high level for both trainings, and the percentage of HR_max_ calculation (based on Nes et al., 2013) showed medium to high training intensities. Furthermore, the ExerCube training yielded significantly better results for flow, enjoyment, and motivation.

### Formula-Based In-Exergame Adaptations

In addition to some relatively unexplored parameters for pre- and in-exergame adaptations (e.g., controller adaptations), the player’s HR has been proposed and explored by various researchers and designers. HR-based adaptation was proved to be a feasible and beneficial approach to balance player’s physical abilities and the exergame challenge and thus to enhance training experience (e.g., engagement, perceived effectiveness, and motivation) and training effects (e.g., performance and outcomes) (Sinclair et al., 2009; Stach et al., 2009; Mueller et al., 2012; Hoffmann et al., 2015; Ketcheson et al., 2015; Martin-Niedecken and Götz, 2016, 2017; Martin-Niedecken, 2018; Martin-Niedecken and Mekler, 2018; Muñoz et al., 2018; Martin-Niedecken et al., 2019a,b). This can be attributed to the fact that in the area of sports and fitness training, HR has already been thoroughly investigated (Ludwig et al., 2018). Furthermore, HR is relatively easy to implement technically in an exergame by means of various wearable devices that allow sending real-time HR data via Bluetooth to the game engine.

So far, only some studies have explored the feasibility of implemented HR prediction models to incorporate HR-based adaptation mechanisms into an exergame (Hoffmann et al., 2015, 2016; Hoffmann and Wiemeyer, 2017b). However, most researchers and designers still rely on age-based formulas to calculate HR_max_ with which to approach and maintain a pre-defined HR range and physical load in exergames. Two examples are given by Robergs and Landwehr (2002) and Nes et al. (2013):

(2) $$H\,R_{\max}=220-\text{age}$$

(3) $$H\,R_{\max}=211-\text{age}\times 0.64$$

These formulas are then implemented in combination with a targeted HR range (defined as percentage of HR_max_) to set the training intensity of an exergame (e.g., beyond 80% of HR_max_ corresponds to high-intensity levels (MacInnis and Gibala, 2017)).

Although numerous validation studies are available (e.g., Nes et al., 2013), these formulas are often criticized because HR and HR_max_ are highly individual parameters that, besides age, can be influenced by various internal (e.g., gender, training status (Nes et al., 2013), genetics (Wang et al., 2009), and mood (Petrov et al., 2014)) and external factors (e.g., environmental conditions, nutrition, and water supply) (Ludwig et al., 2018). Thus, some researchers pre-assessed the individual HR_max_ and targeted range of HR of players with standardized ergometer tests and used this in combination with pre-defined cycling training protocols to implement a pre-defined physical load in a cycling exergame (Barathi et al., 2018; Farrow et al., 2019).

### Exergame-Based Assessments

Another promising approach is the application of exergames as assessment tools (Konstantinidis et al., 2015). This could be another interesting step towards user-friendly and motivating training scenarios and exploiting further in-exergame adaptation mechanisms and strategies. There are hardly any exergame-based assessments to measure cognitive and physical fitness or mental state. Such assessments, although, would allow a more holistic classification of the personal skills, deficits, and mood, and thus would provide a more individualized and detailed default setting of in-exergame adaptation parameters. So far, traditional assessment batteries are still performed manually and outside the (exer)game setting. Thinking about HR_max_ standardized assessments, these testing protocols often involve a player going right up to or even beyond their limits in a way that is not necessarily a pleasant situation to experience. Integrated into an exergame, HR_max_ assessments might become more pleasant since it is known from related work that the immersive and motivating nature of a game helps to shift the focus from one’s own body and the physical strain to the gaming experience and the cognitive level (Martin-Niedecken et al., 2019b). Furthermore, this could increase the overall usability of exergames as attractive and effective training tools on the market, and support trainers and therapists.

## Materials and Methods

To fill the aforementioned gaps, we conducted a comparative study aiming to explore whether there are objective and subjective differences in training intensity (effectiveness) and experience (attractiveness) when the ExerCube is automatically adapted to the targeted HR range (defined as percentage of HR_max_) by either the individually pre-assessed or the formula-based pre-calculated HR_max_. Furthermore, this study aimed to gain some early indications about the usage of the ExerCube to determine individual HR_max_ by comparing the procedure to a standardized ergometer protocol (Maier et al., 2016).

**FIGURE 2:**
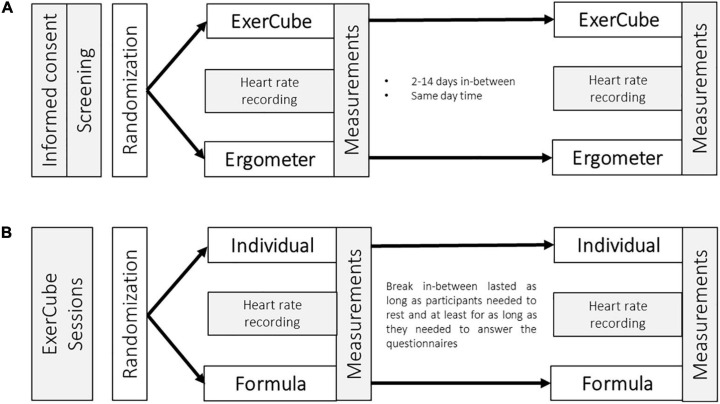
Study procedure. **(A)** Testing sessions to assess individual maximal heart rate. **(B)** Comparison of in-exergame adaptation. In-between **(A)** and **(B)** are 2–14 days.

Because of these objectives, the project consisted of two parts to investigate both study questions (Figure 2). The study ran from November 2019 to February 2020. Measurements were made either at the research group laboratory (ETH Zürich, Hönggerberg, Zurich, Switzerland) or at the Sphery gym (Asylstrasse 64, Zurich, Switzerland). All measurements were made by one investigator who was familiar with the study set-up. Participants were continuously supervised by the experienced investigator and the instructions were provided in a standardized manner for each participant. The ethics committee of the ETH Zürich, Switzerland (EK 2019-N-137), approved the study protocol. Before any measurements were carried out, all eligible participants had to sign a form giving their informed consent according to the Declaration of Helsinki.

### Participants

For this study, the minimal intended sample size of 20 healthy (self-reported by health questionnaire) young adults aged 18–35 years was based on a previous study that examined similar parameters (e.g., HR) (Martin-Niedecken et al., 2020) in the same training setting, as well as on the possibility of losses or refusals. Participants were excluded from the study if one of the following exclusion criteria was presented: (1) history of cardiovascular issues that would prevent training participation, (2) asthma (not controllable), (3) musculoskeletal injuries that would prevent training participation, (4) pain that would be reinforced by sports activities, and (5) pregnancy. For recruitment, different methods were used, such as word-of-mouth and emailing (ETH and company Sphery Ltd, Zurich, Switzerland), without offering any financial compensation for attendance. Prior to the first measurement, all participants were fully informed about the procedure, benefits, and risks of the study.

### Procedure

#### Testing Sessions to Assess Individual Maximal Heart Rate

**FIGURE 3:**
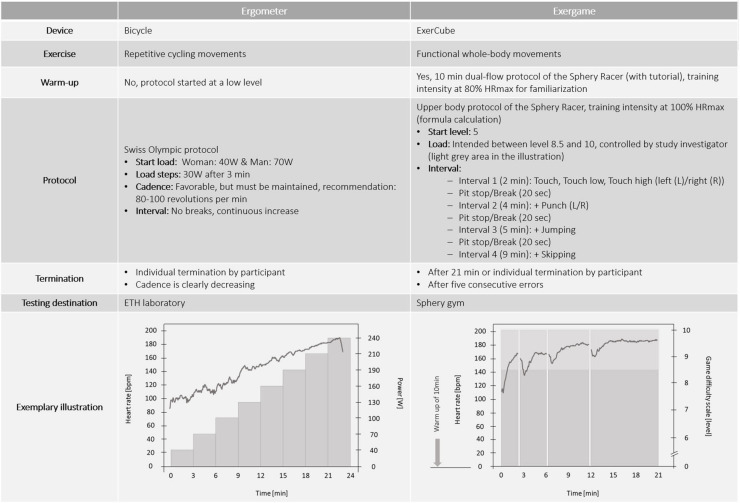
Comparison of the different protocols to determine individual maximal heart-rate. Reference for Swiss Olympic protocol (Maier et al., 2016).

Two separate testing appointments were carried out to assess the individual HR_max_ via an ergometer and an ExerCube protocol. Both testing protocols are presented in Figure 3. At the beginning of the ergometer and the ExerCube session, the resting HR was assessed in a sitting position for 5 minutes (min). HR measurements were continued during the HR_max_ testing sessions. At the end of each session, participants rated their physically and cognitively perceived exertion. The sequence of the testing sessions was randomized to minimize subsequent effects and an intervening period of 2–14 days was set.

#### Comparison of In-Exergame Adaptations

The last appointment included two ExerCube sessions. At the beginning of the appointment, resting HR was assessed in a sitting position for 5 min. HR measurements were continued during both ExerCube sessions. Participants performed the “dual flow protocol” of the Sphery Racer for 11 min (20 seconds (sec) onboarding and calibration scene, 10 min training, and 40 sec pit stops) at a training intensity of 80% HR_max_. The respective HR_max_ of the sessions was defined in two different ways:

• Individual: higher value of both testing sessions
• Formula-based (Nes et al., 2013):

(4) $$H\,R_{\max}=211-\text{age}\times 0.64$$

The ExerCube training protocol started with a short onboarding and calibration scene (20 sec) and contained five exercise intervals on the virtual racing-track, each accessed from an intermediate short pit stop (10 sec).

• Interval 1 (1 min): Touch, Touch low, Touch high (left (L)/right(R))
• Interval 2 (2 min): + Squat, Jumping, Punch (L/R)
• Interval 3 (2 min): + Lunge (L/R)
• Interval 4 (2 min): + Skipping
• Interval 5 (3 min): + Burpee

Exercises started with low-to-moderate intensity (in terms of both physical and cognitive load) and gradually increased over time to high-intensity. The physical and cognitive challenge were gradually adapted independently over the whole training session on a 10-point difficulty scale, where one level was defined as one step on the 10-point scale (e.g., from 5 to 6). In both ExerCube sessions, the game aimed to keep players at 80% HR_max_. A lower HR led to an increase in physical challenge, i.e., speed and exercise frequency (one level per check), while a higher HR led to a decrease (once 100% HR_max_ was reached, this decrease was sped up by three levels to ensure players’ safety). The system employed a strategy for increasing players’ HR, i.e., when 80% HR_max_ has not been reached, it checked actual HR every 20 sec and every 10 sec when HR was above 90% HR_max_. The cognitive challenge increased by one level (resulting in a delayed display of the direction of the next exercise) if the player performed error-free for 20 sec. If the player made three mistakes within 20 sec, the difficulty decreased by one level (resulting in an earlier display of the direction of the next exercise). Thus, physical and cognitive game challenges were controlled each by an independent algorithm. Nevertheless, physical and cognitive load controls were interconnected in the gameplay. For example, when the HR decreased (physical algorithm), the game sped up and the acceleration of the game speed increased the number of exercises and therefore increased the probability of mistakes on the game track (cognitive algorithm).

Both ExerCube sessions were carried out at one appointment. The sequence of the sessions was randomized to balance pre-fatigue effects and minimize subsequent effects. The break in-between lasted as long as the participant needed to rest (self-evaluated) and at least for as long as they needed to answer the questionnaires (about 8 min). After each ExerCube session, the participants rated their general, physically, and cognitively perceived exertion and answered questions about their training experience.

### Assessment

Heart rate data recordings were performed in the resting state, during HR_max_ assessment (ergometer and ExerCube), and during the ExerCube sessions (formula and individual HR_max_) to measure the average HR (HR*_avg_*) and/or HR_max_. Resting HR was determined using the average over the three measurements. For each measurement, resting HR was the average over 5 min under the condition of a steady state being reached before starting the measurement. Participants wore a HR-receiving chest belt of the brand Wahoo (Wahoo Fitness 2014, Atlanta, GA, United States) for HR data collection. The chest belt was either connected (via Bluetooth) to the ExerCube, or the compatible “Wahoo Fitness” App (installed on an android mobile phone).

The Borg 6- to 20-point (6 = very, very light, 20 = very, very hard) and modified 10-point (1 = very weak, 10 = very, very strong) rating scales were selected to assess perceived exertion (Borg, 1982). The 6- to 20-point scale was used to assess the general perceived exertion (Borg) and the 10-point scale was used to assess both physically (Borg*_physical_*) and cognitively (Borg*_cognitive_*) perceived exertion. Training experience was assessed by three questionnaires: (a) Flow Short Scale (FSS), (b) Flow State Scale (FStS), and (c) Physical Activity Enjoyment Scale (PACES). The FSS and the FStS assessed participants’ flow experience (Jackson and Marsh, 1996; Rheinberg et al., 2003). The FSS consists of 13 items and the FStS consists of nine items (short version). In the FSS, the flow experience is measured overall and as three factors: fluency of performance, absorption by activity, and perceived importance. The FStS measures flow experiences during physical activity. Further, participants’ enjoyment of the training was assessed via the PACES, consisting of 18 items (Kendzierski and DeCarlo, 1991; Motl et al., 2001). The FSS and PACES questionnaires were rated on a 7-point Likert scale (FSS: 1 = not at all, 7 = very much; PACES: bipolar statements, 1 = disagree a lot, 7 = agree a lot) and the FStS was rated on 5-point Likert scale (1 = not at all, 5 = very much).

### Analysis

Statistical analysis was conducted in SPSS (IBM SPSS 26). Since the criteria for a parametric analysis were not given, comparisons of the HR values, rating scales, and questionnaires were performed using the Wilcoxon signed-rank test. The level of significance was set at *p* < 0.05. Effect sizes were calculated using the formula (Cohen, 2013):

(5) $$r=\frac{z}{\sqrt{N}}$$

where *z* = z-score and *N* = number of participants.

An effect size 0.1 ≤ *r* < 0.3 is considered a small effect, 0.3 ≤ *r* < 0.5 a medium effect, and *r* ≥ 0.5 a large effect.

## Results

The study was performed with 21 participants (9 women, 12 men) aged 25.3 ± 1.5 years. The participants’ baseline characteristics, fitness status, and exergame experience are presented in Table 1.

**TABLE 1:** Baseline characteristics.

Age (years)	25.3 ± 1.5
Gender	Men: n = 12, Women: n = 9
Years of education	17.4 ± 1.1
Fitness status (self-rated)^1^	3.8 ± 1.0
Activity (hours per week)	3.9 ± 2.8
Resting heart-rate (bpm)	76.5 ± 11.8
Exergame experience	61.9% (yes), 38.1% (no)
ExerCube experience	23.8% (yes), 76.2% (no)

**N* = 21. Data are mean values (standard deviation) as indicated. ^1^Fitness status (self-rated): 1 = poor, 2 = satisfactory, 3 = average, 4 = good, 5 = very good, 6 = competitive sports level.*

### Assessment of Maximal Heart Rate

Results of the testing sessions are presented in Table 2. No significant difference was measured between the ergometer and the ExerCube testing sessions for HR_max_ (*z* = -0.444, *p* = 0.657, *r* = 0.07). Significant higher values for the ExerCube testing session were measured for HR*_avg_* (z = -4.017, *p* < 0.001, *r* = 0.62), time to HR_max_ (*z* = -3.563, *p* < 0.001, *r* = 0.55) and Borg*_cognitive_* (*z* =-3.984, *p* < 0.001, *r* = 0.61). The average training level of the ExerCube was between 8.6 ± 0.5 (mean ± standard deviation) over all participants. The ExerCube testing session was finished by 20 of 21 participants.

**TABLE 2:** Comparison of maximal heart rate testing.

	**Ergometer**	**ExerCube**	**z**	**p**	**r**
HR_max_ (bpm)	192.0 (185.0, 196.0)	189.0 (184.0, 199.0)	–0.444	0.657	0.07
HR_avg_ (bpm)	149.0 (142.0, 156.0)	172.0 (165.0, 181.0)	–4.017	<0.001*	0.62
Time to HR_max_ (min)	19.1 (17.2, 24.7)	16.1 (15.2, 19.1)	–3.563	<0.001*	0.55
Borg cognitive (1–10)	2.0 (1.0, 2.5)	6.0 (5.0, 6.0)	–3.984	<0.001*	0.61
Borg physical (1–10)	8.0 (8.0, 9.0)	8.0 (7.0, 9.0)	–1.067	0.286	0.16

*N* = 21. Data are median (interquartile range) as indicated. Data comparison was analyzed using Wilcoxon-signed rank test. *p < 0.05, *p*-values are two tailed. Effect size *r*: 0.1 ≤ *r* < 0.3 small effect, 0.3 ≤ *r* < 0.5 medium effect, *r* ≥ 0.5 large effect. HR_max_ = maximal heart rate, HR_avg_ = average heart rate.

### Training Experience Comparing Individual Versus Formula HR_max_

One participant had to be excluded from the analysis due to technical difficulties. No significant differences were measured for HR and perceived exertion parameters (Table 3). For the questionnaire data, a significant difference resulted for the item “unambiguous feedback” in favor of the individual HR_max_ condition (*z* = -2.121, *p* = 0.034, *r* = 0.34). All the other questionnaire data showed no significant differences. Results of the questionnaire data are presented in Table 4. Table 5 shows an overview of the perceived feelings between the formula and the individual HR-based ExerCube sessions.

**TABLE 3:** Comparison of ExerCube conditions (formula vs. individual) for heart rate and perceived exertion.

	**Formula**	**Individual**	***z***	***p***	***r***
Pre-defined HR_max_ (bpm)	195.0 (194.5, 195.6)	193.0 (186.8, 200.8)	–0.766	0.444	0.12
HR_max_ (bpm)	178.0 (170.5, 187.5)	181.0 (171.0, 185.0)	–0.065	0.948	0.01
HR_avg_ (bpm)	151.0 (141.3, 157.3)	150 (139.3, 163.0)	–0.497	0.619	0.08
Borg (6–20)	14.0 (13.0, 15.0)	15.0 (13.0, 15.0)	–1.035	0.301	0.16
Borg cognitive (1–10)	4.0 (3.0, 5.0)	4.0 (3.0, 5.0)	–1.071	0.284	0.17
Borg physical (1–10)	5.0 (4.3, 6.0)	5.5 (5.0, 6.0)	–0.263	0.793	0.04

*N* = 20. Pre-defined HR_max_ was used to determine training intensity (defined as percentage of HR_max_) for in-exergame adaptation. Data are median (interquartile range) as indicated. Data comparison was analyzed using Wilcoxon-signed rank test. *p < 0.05. *p*-values are two tailed. Effect size *r*: 0.1 *≤ r* < 0.3 small effect, 0.3 ≤ *r* < 0.5 medium effect, *r* ≥ 0.5 large effect. HR = heart rate, HR_max_ = maximal heart rate, HR_avg_ = average heart rate.

**TABLE 4:** Comparison of ExerCube conditions (formula vs. individual) for enjoyment and flow.

	**Formula**	**Individual**	***z***	***p***	***r***
**PACES**	6.0 (5.3, 6.3)	5.9 (5.3, 6.4)	–0.047	0.962	0.01

**FSS**	5.6 (4.9, 6.2)	5.9 (5.2, 6.1)	–0.468	0.640	0.07
Absorption	5.5 (5.3, 6.3)	5.6 (5.3, 6.0)	–0.263	0.793	0.04
Fluency	5.6 (4.8, 6.1)	5.8 (5.3, 6.3)	–0.787	0.431	0.12
Perceived importance	2.5 (2.0, 3.3)	2.3 (1.8, 3.6)	–0.682	0.495	0.11

**FStS**	4.3 (3.9, 4.5)	4.3 (4.0, 4.6)	–0.969	0.333	0.15
Challenge-skill balance	5.0 (4.3, 5.0)	5.0 (5.0, 5.0)	–1.414	0.157	0.22
Action-awareness merging	4.5 (3.0, 5.0)	4.0 (4.0, 5.0)	–0.758	0.449	0.12
Clear goals	5.0 (4.0, 5.0)	5.0 (5.0, 5.0)	–1.150	0.132	0.18
Unambiguous feedback	4.0 (3.0, 5.0)	4.0 (4.0, 5.0)	–2.121	0.034*	0.34
Concentration on the task	5.0 (4.0, 5.0)	5.0 (4.0, 5.0)	–1.134	0.257	0.18
Sense of control	4.0 (3.0, 4.8)	4.0 (3.0, 4.0)	–0.535	0.539	0.08
Loss of self-control	5.0 (5.0, 5.0)	5.0 (5.0, 5.0)	–1.414	0.157	0.22
Transformation of time	4.0 (4.0, 5.0)	4.0 (3.3, 5.0)	–1.732	0.083	0.27
Autotelic experience	4.0 (3.3, 5.0)	4.0 (3.0, 5.0)	–0.905	0.366	0.14

*N* = 20. Data are median (interquartile range) as indicated. Data comparison was analyzed using Wilcoxon-signed rank test. *p < 0.05. *p*-values are two tailed. Effect size *r*: 0.1 ≤ *r* < 0.3 small effect, 0.3 ≤ *r* < 0.5 medium effect, *r* ≥ 0.5 large effect.

**TABLE 5:** Survey of different perceived feelings between formula and individual session.

	**Formula**	**Individual**	**No difference**
Which session was more exhausting?	*n* = 11 (55%)	*n* = 7 (35%)	*n* = 2 (10%)
Which session felt more pleasant?	*n* = 9 (45%)	*n* = 8 (40%)	*n* = 3 (15%)

**N* = 20.*

## Discussion

The aim of this study was to explore whether there are objective and subjective differences in training intensity (effectiveness) and experience (attractiveness) when the ExerCube is automatically adapted to the targeted HR range (defined as percentage of HR_max_) by the individually pre-assessed or formula-based pre-calculated HR_max_. Furthermore, this study aimed to gain some early indications about the usage of the ExerCube to determine individual HR_max_ by comparing the procedure to a standardized ergometer protocol. The following sections discuss the results in the context of related work and knowledge in the area of (individualized) in-exergame adaptations and exergame-based assessment using HR. Furthermore, implications are illustrated for future adaptive exergame design and research topics.

### In-Exergame Adaptations: Training Experience and Intensity Comparing Individual and Formula Based HR_max_

Comparing the individual and formula-based HR_max_ ExerCube conditions, one significant difference was assessed for the item “unambiguous feedback” in favor of the individual HR_max_ condition. This significant difference, however, must be considered with caution since a significant difference does not always imply a (clinically) relevant difference (Page, 2014). Furthermore, this questionnaire item was already at a high level for both conditions.

All the other questionnaire items, the short survey on player’s feelings, and the assessed subjective and objective training intensity showed favorable values independently of the HR_max_ condition. The reason for this might be that the comparison of the pre-defined individual HR_max_ values (formula and individual), used for defining the training intensity in the ExerCube, revealed no significant difference, indicating that the ExerCube allowed a reliable in-exergame adaptation as well as gameplay experience. These positive experiences, including high feelings of flow and enjoyment, as well as the favorable training intensity are in line with previous ExerCube studies (Martin-Niedecken and Mekler, 2018; Martin-Niedecken et al., 2019a,b). Thus, in-exergame adaptations via HR seem to be feasible for triggering an individually attractive and effective gameplay experience, as demonstrated in previous studies (Stach et al., 2009; Mueller et al., 2012; Hoffmann et al., 2015; Martin-Niedecken and Götz, 2016, 2017; Martin-Niedecken, 2018; Muñoz et al., 2018).

Furthermore, findings of this study indicate that the formula concept may be a good alternative to the individually determined HR_max_ for in-exergame adaptation. Nevertheless, the presented study included a rather homogeneous group of young adults, resulting in an individual HR_max_ that was well covered by the formula. A huge fitness study, however, showed that the implemented formula adequately explained HR_max_ by age, considering an age range of 19–89 years (Nes et al., 2013). Thus, the formula concept might also be suitable for older age groups. Nontheless, one must consider that the formula is an approximation of the real value and (maximal) HR can (daily) be influenced by various internal and external factors such as gender, circadian cycle, blood pressure, lifestyle factors, physical activity, and mental status (Londeree and Moeschberger, 1984; Valentini and Parati, 2009). Therefore, this study provided some early indications that have to be substantiated with more studies considering different player attributes (e.g., activity levels and mental status). Furthermore, it might be interesting to explore which application area (e.g., rehabilitation, fitness, and prevention) might benefit the most from a HR-based training.

### Exergame-Based Assessment: ExerCube as a HR_max_ Assessment Tool

In terms of HR_max_ assessment, results demonstrated that both testing protocols triggered comparable HR_max_ as no significant difference was present. This result gave a first impression that it seems feasible to assess individual HR_max_ using the ExerCube. To our best knowledge, this study is one of the first investigations that assessed individual HR_max_ using an exergame.

Furthermore, results showed a significantly higher HR*_avg_* and significantly shorter time to reach HR_max_ for the ExerCube assessment protocol. This might be explained by the fact that both protocols had different design attributes. The ExerCube started directly with a fast increase of the intensity and the warm-up was not part of the testing protocol. Interestingly, 20 out of 21 participants finished the whole ExerCube session lasting 21 min, even if the HR_max_ was reached before the protocol ended. This circumstance might be due to the fact that the ExerCube was not only a physical challenge, but also stimulated cognitive processes that presented in a significantly higher cognitive load for the ExerCube training protocol. In combination with the previously decribed game flow and enjoyment, this higher cognitive engagement might have distracted the player’s focus away from their physical exhaustion and therefore let participants perform longer on a high intensity (Bertollo et al., 2015; Bigliassi et al., 2016). It may be that the participants could have done their testing in the ExerCube even longer at this high level as no participant was stopped because of continuous performance errors.

Overall, the results of this comparison led to several observations that might be useful concerning exergaming as HR_max_ assessment. A familiarization phase seems to be mandatory for exergames, as conventional HR_max_ assessments usually have a less complex environment, exercise performance, and/or movement patterns, respectively. This familiarization is an important control process to ensure that overexertion is not caused by incorrect performance or misunderstanding of the performance. The length of this phase should be determined depending on the complexity of the exergame, ensuring that the participant has understood the exergame control and play mechanism. A warm-up phase could be used for familiarization, and therefore precede the testing session. Still, a warm-up phase can also be included in the assessment, as is usually the case for conventional HR_max_ testing protocols, by starting at a low-intensity level. Regarding termination criteria, overexertion can be determined by the participant (subjective), as in conventional performance tests or, particularly for exergames, by a pre-defined number of failures, movement precision, accuracy, and power (objective) or by performance worsening (subjective). Moreover, these overexertion parameters as well as the familiarization and warm-up phase are important precautions to ensure the safety of participants.

A special feature of exergames is the unique combination of physical, cognitive, and mental load. The nature of HR_max_ testing protocols is to increase the HR via high physical load. In the ExerCube protocol, the physical load was increased by exercise frequency (racing speed) and physically intensive exercises (e.g., skipping). Furthermore, the ExerCube included cognitive stimuli via information processing of the virtual track and the required in-game actions (e.g., reaction, planning, and coordination). In this study, the cognitive load of the ExerCube was more or less at the same level throughout the testing session. Cognitive stimulation, as mentioned before, could be supportive as it might distract from the physical exhaustion (Bertollo et al., 2015; Bigliassi et al., 2016). However, an overloaded cognitive stimulation could have opposite effects as fewer resources might be available for the physical performance (MacMahon et al., 2014). On the other hand, an increasing cognitive load could also be a part of a HR_max_ testing protocol as a high cognitive load seems to increase HR (Mehler et al., 2009; Rudolf et al., 2016). Next to physical and cognitive load, mental load can also have an effect on the HR as excitement and stress can initiate biological responses (Valentini and Parati, 2009). Nevertheless, how physical, cognitive, and mental load should be combined in a HR_max_ testing protocol is part of future studies because further research is needed to understand, strengthen, and complement the interaction of these loads.

### Parameters for Real-Time In-Exergame Adaptations

These study results initiate the discussion of how HR or further parameters (e.g., insights from eye tracking) could be used (exclusively or in combination) for real-time in-exergame adaptation, allowing an individually tailored exergame experience. Individual training and game adaptations based on user requirements may increase training/gaming motivation (attractiveness) and success (effectiveness) (Sinclair et al., 2007, 2009; Martin-Niedecken and Götz, 2016, 2017; Martin-Niedecken, 2018; Martin-Niedecken and Mekler, 2018; Martin-Niedecken et al., 2019a,b).

Knowledge from different research fields and disciplines (e.g., sport science and HCI) should be used to examine different parameters assessing physical (Wallace et al., 2014; Burgess, 2017; Coyne et al., 2018; McLaren et al., 2018), cognitive (Solovey et al., 2014; Grassmann et al., 2016; Oschlies-Strobel et al., 2017; He et al., 2019; Hughes et al., 2019; Zhou et al., 2020), and mental (Schrader et al., 2017; Mostefai et al., 2019) load. Objective parameters could be defined by physiological and performance-related factors (Vasilyev et al., 2019). Physiological parameters could be measurements, outcomes, and variables related to HR (e.g., heart rate variability), respiration, eye tracking, facial expression, skin conductance, and brain (e.g., functional near-infrared spectroscopy and electroencephalogram) and muscle (e.g., electromyography) activity. Performance-related parameters could be reaction time and failure rate as well as movement execution, acceleration, deceleration, and accuracy. In addition to the objective parameters, subjective parameters (e.g., rating scales) could be used to determine the different loads (Smith et al., 2014; Saw et al., 2016; Meckel et al., 2018).

An essential starting point is that the parameters should suit the exergame mechanics, components, and (training) goal. Exergames could record performance-related data but also integrate devices or game mechanics that measure and assess objective (e.g., physiological and performance-related factors) and subjective (e.g., rating scales) parameters to determine the player’s physical, cognitive, and mental load. In real-time in-exergame adaptations, the parameters can be implemented at different levels of the exergame such as controller (e.g., sensitivity of tracking), game (e.g., audio-visual appearance) and player (e.g., range of motion) (Martin-Niedecken and Götz, 2017; Martin-Niedecken, 2018; Martin-Niedecken et al., 2019a; Wiemeyer, 2019). However, due to the complex combination of physical, cognitive, and mental components, further conceptual thoughts have to be considered in future studies.

Studies that look at the individual components of an exergame and their interdependencies seem promising to determine the influence of the above-mentioned loads and thus to support the integration of suitable objective and subjective parameters for real-time in-exergame adaptations (Gutjahr et al., 2019; Martin-Niedecken et al., 2020). For example, one should keep in mind whether the game scenario, and thus the processed information, is coupled (e.g., running in an engaging environment and catching a robber) or uncoupled (e.g., running and solving calculation tasks) to the player’s physically performed movements because this further affects the interplay of the exergame components (Herold et al., 2018; Martin-Niedecken et al., 2019a).

Exergame-based assessments might support the determination of parameter(s) under a maximal (e.g., HR_max_), optimal or standardized testing situation. Result(s) of performance tests can then be used to individually determine the starting load and/or the individually targeted training intensity (e.g., 80% HR_max_), and that in turn can be used to control real-time in-exergame adaptations (Hardy et al., 2015). These individual in-exergame adaptations can be controlled by specific algorithms and may even be improved by the inclusion of artificial intelligence. By requiring and storing player information in internal models, AI might allow dynamic modeling and prediction of an exergame track (Wenger, 2014; Streicher and Smeddinck, 2016; Hoffmann and Wiemeyer, 2017a; Ludwig et al., 2018; Gang et al., 2019).

Overall, the usability and feasibility of these parameters have to be considered in proportion to the potential gain for the exergame’s attractiveness and effectiveness.

### Limitations

In the context of this study, some limitations have to be mentioned. One is the homogeneous study population of fairly fit younger adults, allowing only a limited generalization of the study results as HR in particular is an individual parameter that depends on several internal and external factors. Therefore, future studies should consider including participants from different age ranges and different fitness levels to check whether these results can be replicated or if different considerations or further adjustments have to be made for different conditions.

Furthermore, two limitations in the context of the HR_max_ testing session should be mentioned. The testing protocols used differed in their structure and this might have influenced the study results. Nevertheless, the study results gave early indications of how far the ExerCube could already be used to determine HR_max_ in its existing design. Moreover, the speed of the ExerCube during the HR_max_ testings was subjectively regulated (maximal performance for each participant) by the observing study investigator, allowing maximal speed adaptation that would not otherwise be possible due to automatic in-exergame adaptation of the ExerCube. A next step would be to elaborate standardized testing protocol(s) for the ExerCube to regulate the subjective components down to a minimum.

In addition, the use of a treadmill instead of a bicycle might be even more appropriate since the movements in the ExerCube were performed in a standing position. Nevertheless, the difference of HR_max_ between bicycle and treadmill ergometer tend to be small at young ages but become larger with increasing age (Londeree and Moeschberger, 1984). Furthermore, this testing protocol already gave an early indication of how the ExerCube can be used to assess HR_max_.

## Conclusion

Given the urgent demand for attractive and effective training tools, exergames represent a promising and innovative approach. Nonetheless, exergames need to fulfill certain design and training aspects to be a real alternative to conventional training methods. A promising approach towards boosting the effectiveness and attractiveness of exergames is the personalization through in-exergame adaptations. Among other things, HR has often been used to balance the physical load and training intensity of exergames with the player’s fitness skills. The most common way was to implement an age-based HR_max_ formula into the exergame, allowing the exergame to reach a targeted training intensity (e.g., percentage of HR_max_). To contribute to this promising topic, we explored different HR conditions (a standardized individually pre-assessed HR_max_ and a formula-based pre-calculated HR_max_) in the exergame ExerCube in healthy young adults and compared the impact on training intensity and experience. Comparing the results of the two conditions, no significant differences were found for HR parameters and perceived exertion (physical and cognitive), nor for overall flow feelings and enjoyment. Thus, the formula-based in-exergame adaptation approach was suitable in the presented study population, and the ExerCube provided an equally reliable in-exergame adaptation and comparable exergame play experience. Furthermore, we investigated the usage of an ExerCube protocol to determine HR_max_ by comparing the procedure to a standard ergometer protocol. Results indicated that the ExerCube seems to be a feasible tool for assessing individual HR_max_. Finally, we derived some implications for future adaptive exergame design and research topics.

## Data Availability Statement

The raw data supporting the conclusions of this article will be made available by the authors, without undue reservation.

## Ethics Statement

The studies involving human participants were reviewed and approved by the ethics committee of the ETH Zürich, Switzerland (EK 2019-N-137). The participants provided their written informed consent to participate in this study. Written informed consent was obtained from the individual(s) for the publication of any potentially identifiable images or data included in this article.

## Author Contributions

AM-N and AS conceptualized, designed, and drafted the manuscript. TS conducted the study (supervised by AS and AM-N). TS and AS led the data analysis and interpretation. AM-N also contributed to the latter. All authors created the study design, compiled the training protocols, carefully selected the assessment methods, and critically reviewed and approved the final manuscript.

## Conflict of Interest

The authors declare that the research was conducted in the absence of any commercial or financial relationships that could be interpreted as a potential conflict of interest. Besides their academic career, AM-N and AS are also working for Sphery. AM-N is the Co-Founder and CEO of the startup company Sphery Ltd., that developed the ExerCube. AS has been working as Senior Research and Development Manager at Sphery since November 2019. No revenue was paid (or promised to be paid) directly to AM-N, to AS, to Sphery or the research institutions. The remaining author declares that the research was conducted in the absence of any commercial or financial relationships that could be construed as a potential conflict of interest.

## Abbreviations

HR: heart rate
R&D: research and development
HR_max_: maximum heart rate
HR_avg_: average heart rate
fHIIT: functional high-intensity interval training
HCI: human-computer interaction
min: minutes
sec: seconds.
